# The Multidirectional Auxeticity and Negative Linear Compressibility of a 3D Mechanical Metamaterial

**DOI:** 10.3390/ma13092193

**Published:** 2020-05-10

**Authors:** Krzysztof K. Dudek, Daphne Attard, Ruben Gatt, James N. Grima-Cornish, Joseph N. Grima

**Affiliations:** 1 Institute of Physics, University of Zielona Gora, ul. Szafrana 4a, 65-069 Zielona Gora, Poland; 2 Metamaterials Unit, Faculty of Science, University of Malta, MSD 2080 Msida, Malta; ruben.gatt@um.edu.mt (R.G.); james.n.grima-cornish@um.edu.mt (J.N.G.-C.); joseph.grima@um.edu.mt (J.N.G.); 3 Department of Chemistry, Faculty of Science, University of Malta, MSD 2080 Msida, Malta

**Keywords:** auxetic, negative compressibility, mechanical metamaterials

## Abstract

In this work, through the use of a theoretical model, we analyse the potential of a specific three-dimensional mechanical metamaterial composed of arrowhead-like structural units to exhibit a negative Poisson’s ratio for an arbitrary loading direction. Said analysis allows us to assess its suitability for use in applications where materials must be able to respond in a desired manner to a stimulus applied in multiple directions. As a result of our studies, we show that the analysed system is capable of exhibiting auxetic behaviour for a broad range of loading directions, with isotropic behaviour being shown in some planes. In addition to that, we show that there are also certain loading directions in which the system manifests negative linear compressibility. This enhances its versatility and suitability for a number of applications where materials exhibiting auxetic behaviour or negative linear compressibility are normally implemented.

## 1. Introduction

Mechanical metamaterials [1,2] are rationally designed systems that are capable of exhibiting unusual mechanical properties. Over the the last thirty years, this class of materials has attracted a lot of attention in the scientific community, which stems from the continuously growing number of applications wherein such systems can prove their usefulness. In fact, mechanical metamaterials have been reported to improve the efficiency of a plethora of devices, including soundproofing [3,4,5], biomedical (stents, skin grafts, smart dressings and implants) [6] and protective devices [7,8,9,10]. In recent years, these systems have also been applied to damping mechanisms (car bumpers, seismic protection of buildings) [11,12] and modern sports equipment [13] and have the potential to manifest an enhanced mechanical response in comparison to their conventional counterparts. This versatility of mechanical metamaterials is possible due to a number of different counter-intuitive mechanical properties that they can exhibit depending on the manner in which they are designed. It seems that the most pursued of such mechanical properties is the negative Poisson’s ratio (auxetic behaviour) [7,14,15,16,17,18,19,20,21,22,23] which characterises bodies that can expand laterally when uniaxially stretched and conversely contract laterally when uniaxially compressed. Some of the other highly-desired, unusual mechanical properties are negative stiffness [24,25,26,27,28], negative compressibility [29,30,31,32,33,34,35] and negative thermal expansion [36,37].

Mechanical metamaterials can be designed in a number of ways allowing them to assume an abundance of spatial configurations. However, as the first studies focused on these systems were emerging, they were primarily devoted to two-dimensional metamaterials [38,39,40], which was up to an extent motivated by their relative simplicity and the ease with which they could be manufactured. Nevertheless, in many modern applications, the system must be able to manifest a desired mechanical response upon being subjected to a stimulus applied from multiple spatial directions. Hence, scientists devoted a lot of attention to studies related to three-dimensional mechanical metamaterials capable of exhibiting auxetic behaviour and other unusual mechanical properties. In fact, different examples of 3D mechanical metamaterials can be found at many different scales, including the microscale [41,42] where it has been shown that they can be used to design auxetic structures [43,44] and ultrastiff lattices [45] which can manifest a number of other interesting phenomena. Some examples of such structures correspond to materials capable of twisting upon being subjected to an external stimulus [46,47], structures exhibiting negative acoustic indices [48] and materials with negative thermal expansion [49]. Recently it was also reported that such systems can manifest programmable behaviour [50]. In addition to the 3D mechanical metamaterials constructed at the microscale, there is also a broad range of studies devoted to such systems constructed at the macroscale, where they can be conveniently manufactured by means of standard 3D printers. Some of the most interesting directions of studies wherein such structures can be utilised include highly stretchable materials [51,52], auxetic materials [53,54,55,56,57] and materials exhibiting efficient energy absorption [26,58,59,60,61].

Despite all of the studies related to 3D mechanical metamaterials at different scales, more work is still being done, and is yet to be done to design novel types of such systems which should be capable of exhibiting unusual mechanical properties, such as auxetic behaviour. It is also possible to discover new properties of the already reported 3D mechanical metamaterials which have not been investigated in the depth required to have a full understanding of their behaviour. In fact, a very good example of such an approach is the recent work [28] inspired by the famous two-dimensional arrowhead system [62] wherein a novel 3D magneto-mechanical metamaterial was proposed that is capable of exhibiting negative stiffness and auxetic behaviour at the same time. This study also follows the work by Lim [63] wherein a 3D arrowhead-based system was analysed for its auxetic behaviour manifested for loading in axial directions and the work by Chen et al. [64] where a similar system was analysed from the perspective of deformation in a specific axial direction. However, aspects which still need to be further addressed are mechanical properties of such systems in directions other than main axial ones, and the analysis of its potential to exhibit unusual mechanical behaviour other than auxetic behaviour, with one such example being negative linear compressibility (NLC). Such studies could allow one to determine how this system would respond to a stimulus applied in an arbitrary direction—information which is important to successfully apply it to the industry. In view of this, in this work, we are going to analyse Poisson’s ratio and linear compressibility properties of a non-magnetic equivalent of the aforementioned structure for various loading directions.

## 2. Methods

### 2.1. Geometry

**Figure 1:**
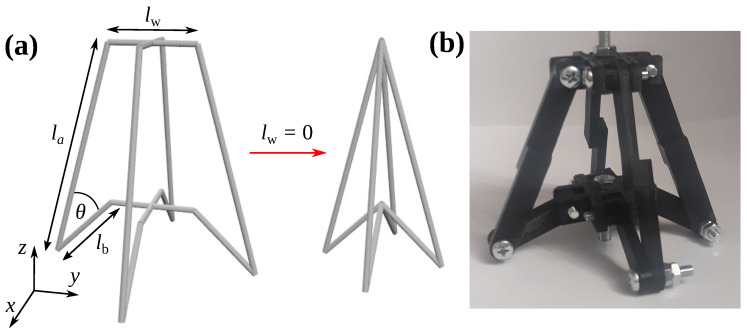
Panels show (**a**) a single unit-cell of the considered model in the case when $l_{w}\neq 0$ and $l_{w}\to 0$, and (**b**) an example of the 3D-printed prototype of the unit-cell.

In this work, we analysed the mechanical properties for different loading directions of a 3D mechanical metamaterial proposed recently in [63] and modified by the addition of magnetic inclusions enabling it to manifest multiple counter-intuitive mechanical propeprties at the same time in [28]. The considered system, with its unit-cell being shown schematically in Figure 1, consists of structural units composed of two orthogonal planes resembling structural units of the so-called arrowhead system [62]. Each of such planes is constructed by means of three pairs of rigid ligaments having lengths denoted as $l_{a}$, $l_{b}$ and $l_{w}$, where $l_{a}>l_{b}$. The angle between ligaments having lengths of $l_{a}$ and $l_{b}$ that form the same structural unit is denoted as θ. In this work, it was assumed that this angle is the sole parameter required to describe the deformation of the entire system. This approach is justified if the ligaments of length $l_{w}$ remain parallel throughout the deformation process or if $l_{w}$ is small in comparison to $l_{a}$ and $l_{b}$. In fact, in this study, in order to determine the analytical expressions describing the mechanical properties of the system, it was assumed that $l_{w}\to 0$ to satisfy this condition (see Figure 1a).

**Figure A1:**
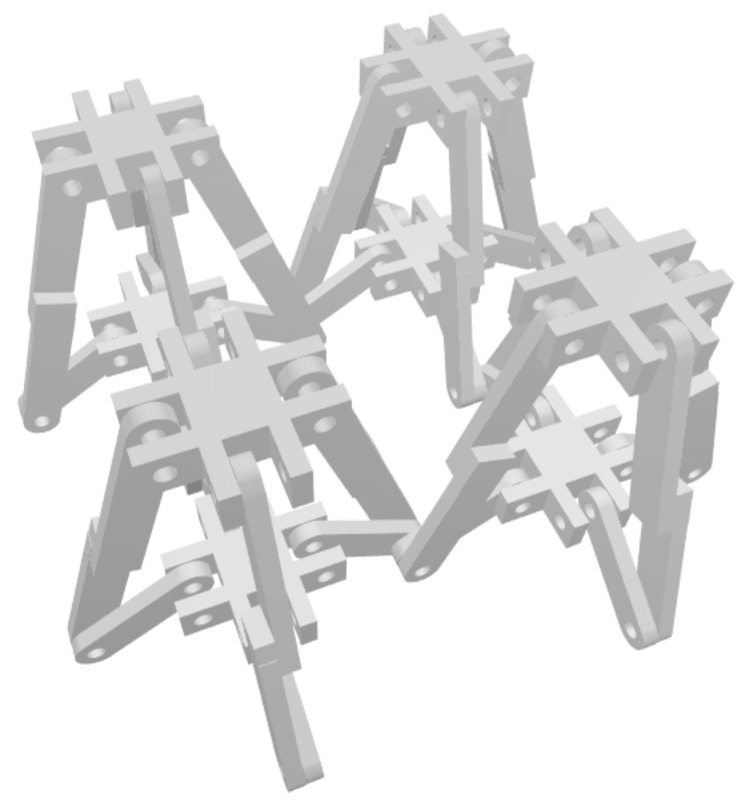
A diagram showing a system studied in this work made from a 2×2×1 assembly of structural units.

It is important to emphasise the fact that the considered system is not a purely hypothetical concept, and it can be conveniently constructed by means of different techniques. In fact, as shown in Figure 1b, it can be achieved by appropriately designing the system in a way where different ligaments would be connected to each other in a pin-jointed manner. It is also worth noting that the design of the proposed prototype allows for the easy construction of a larger system composed of similar structural units connected to each other as shown in Appendix A (see Figure A1).

**Figure 2:**
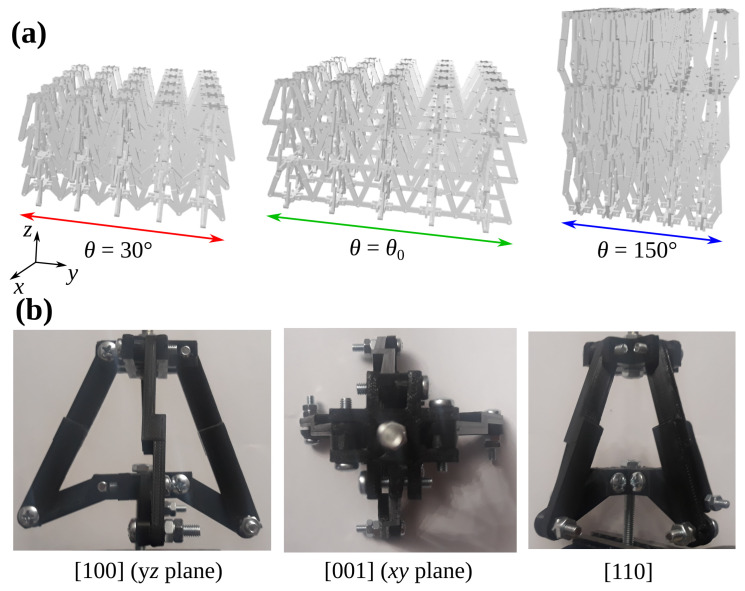
Panels show (**a**) an example of how the considered system would look at different configurations associated with a change in θ should it be constructed by means of structural units like the one shown in Figure 1b, and (**b**) projections of the unit-cell of the proposed prototype in different planes.

Upon closer inspection of the considered model, it may be noted that its projections in different planes formed by principal axes and other planes, may result in relatively simple shapes. More specifically, as shown schematically in Figure 2b, the projection of a single unit-cell in the xz and yz planes closely resembles an arrowhead. In fact, the projection of the structure in these two planes is exactly the same. On the other hand, the projection of the unit-cell in the xy plane is always a square irrespective of the value of θ. This means that one should expect an isotropic behaviour for loading in any direction in this plane. Together with the symmetry of the system, it is also indicative of the fact that linear dimensions of the unit-cell in the *x* and *y* directions are identical. In fact, linear dimensions of the unit-cell can be defined as follows [28]:

(1) $$L_{x}=L_{y}=l_{w}+\frac{2l_{a}l_{b}\sin\theta }{\sqrt{l_{a}^{2}+l_{b}^{2}-2l_{a}l_{b}\cos\theta }}\;\;L_{z}=\sqrt{l_{a}^{2}+l_{b}^{2}-2l_{a}l_{b}\cos\theta }\;.$$

### 2.2. Deformation Process

Deformation of the considered system can be described by a change in the value of θ. In fact, upon changing the extent of this angle, one can note that the structure may assume one of the two characteristic types of configurations where $\theta <\theta_{0}$ or $\theta >\theta_{0}$. The transition angle $\theta_{0}$ used in the above definition can simply be determined by means of the following expression: $\theta_{0}=\arccos\left(l_{b}l_{a}^{-1}\right)$. It is also worth noting that in the case where $\theta <\theta_{0}$, the projection of the system in the xz and yz planes resembles the auxetic two-dimensional system. In fact, based on Figure 2a, it may be noted that the entire system manifests auxetic behaviour in these two planes for loading along the *z*-axis. Conversely, for the same loading direction, the system contracts laterally in the two aforementioned planes.

### 2.3. Mechanical Properties

**Figure 3:**
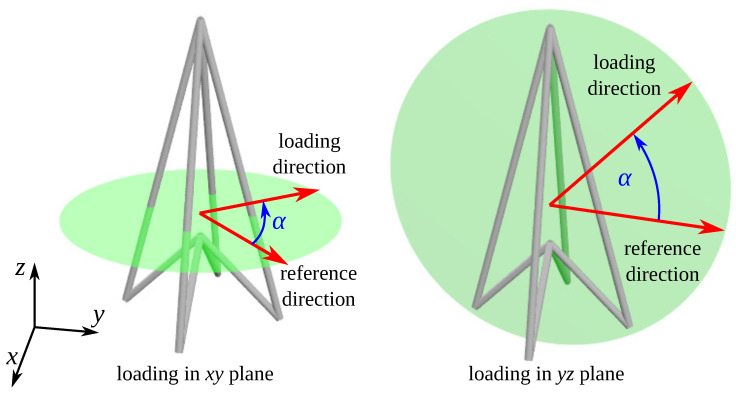
Diagram portraying xy and yz planes in which different uniaxial loading directions were considered. Note that the “reference” direction of loading which is being used to calculate the value of α should be adjusted depending on the particular mechanical property; e.g., it should be rotated by $90^{\circ }$ around the *y* axis to obtain $\nu_{xz}$ from $\nu_{zx}$, etc. Additionally, to denote the Poisson’s ratio for loading in the arbitrary direction for example in the xy plane, the notation $\nu_{xy}\left(\alpha \right)$ is used which indicates that the loading and transverse deformation directions are defined in the xy plane but are rotated by the angle α with respect to the *x* and *y* axes respectively.

In this work, we are interested in assessing the potential of the considered system to exhibit unusual mechanical properties for different loading directions. More specifically, the focus was on the possibility of observing negative Poisson’s ratios and NLC for an arbitrary deformation direction in the planes formed by principal axes; i.e., xy, xz and yz planes (see Figure 3). In order to do this, we are going to use the approach proposed elsewhere [39,40,65]. According to this approach, before deriving mechanical properties such as the Poisson’s ratio in an arbitrary direction within the aforementioned planes, it is necessary to first determine the mechanical properties of the structure along the principal axes. The first of the considered mechanical properties, i.e., Poisson’s ratio, is commonly defined as [1]:

(2) $$\begin{array}{c} \nu_{xy}=\frac{1}{\nu_{yx}}=-\frac{\varepsilon_{y}}{\varepsilon_{x}}=-\frac{L_{x}}{L_{y}}\left(\frac{dL_{y}}{d\theta }\right){\left(\frac{dL_{x}}{d\theta }\right)}^{-1}\\ \nu_{xz}=\frac{1}{\nu_{zx}}=-\frac{\varepsilon_{z}}{\varepsilon_{x}}=-\frac{L_{x}}{L_{z}}\left(\frac{dL_{z}}{d\theta }\right){\left(\frac{dL_{x}}{d\theta }\right)}^{-1}\\ \nu_{zy}=\frac{1}{\nu_{yz}}=-\frac{\varepsilon_{y}}{\varepsilon_{z}}=-\frac{L_{z}}{L_{y}}\left(\frac{dL_{y}}{d\theta }\right){\left(\frac{dL_{z}}{d\theta }\right)}^{-1} \end{array}$$

where $L_{x}$, $L_{y}$ and $L_{z}$ stand for the already defined dimensions of the unit-cell. After substituting expressions defining these dimensions into Equation (2), one can obtain the following:

(3) $$\nu_{xy}=-1$$

(4) $$\nu_{xz}=\frac{\frac{1}{2}\left(2l_{a}l_{b}\sin\left(\theta \right)+l_{w}\sqrt{l_{a}^{2}+l_{b}^{2}-2l_{a}l_{b}\cos\left(\theta \right)}\right)\sqrt{l_{a}^{2}+l_{b}^{2}-2l_{a}l_{b}\cos\left(\theta \right)}\sin\left(\theta \right)}{l_{a}l_{b}\sqrt{l_{a}^{2}+l_{b}^{2}-2l_{a}l_{b}\cos\left(\theta \right)}\sin^{2}\left(\theta \right)-{\left(l_{a}^{2}+l_{b}^{2}-2l_{a}l_{b}\cos\left(\theta \right)\right)}^{1.5}\cos\left(\theta \right)}$$

(5) $$\nu_{zy}=\frac{2\left(l_{a}l_{b}\sqrt{l_{a}^{2}+l_{b}^{2}-2l_{a}l_{b}\cos\left(\theta \right)}\sin^{2}\left(\theta \right)-{\left(l_{a}^{2}+l_{b}^{2}-2l_{a}l_{b}\cos\left(\theta \right)\right)}^{1.5}\cos\left(\theta \right)\right)}{\left(2l_{a}l_{b}\sin\left(\theta \right)+l_{w}\sqrt{l_{a}^{2}+l_{b}^{2}-2l_{a}l_{b}\cos\left(\theta \right)}\right)\sin\left(\theta \right)\sqrt{l_{a}^{2}+l_{b}^{2}-2l_{a}l_{b}\cos\left(\theta \right)}}.$$

It is worth noting that $\nu_{xy}=\nu_{yx}=-1$ which stems from the fact that $L_{x}=L_{y}$.

In addition to the calculation of the Poisson’s ratio for loading in the directions corresponding to principal axes, we are also interested in deriving similar expressions corresponding to the linear compressibility. However, there is a significant difference between the two properties. Namely, the Poisson’s ratio depends solely on relative dimensions of the system (it is scale independent) and not on the properties of the material which it is made of. On the other hand, in the case of the linear compressibility, the material’s properties must be taken into account. In order to do that, we employ the energy-based approach where we assume that the unit-cell has a certain stiffness associated with it which corresponds to twelve hinges that must be rotated in order to deform the system [66]. In this case, the total work associated with an infinitesimal deformation of the unit-cell can be defined as:

(6) $$W=12\left[\frac{1}{2}K_{h}{\left(d\theta \right)}^{2}\right]$$

where $K_{h}$ is the stiffness constant associated with a single hinge. Thus, as a result of the conservation of energy principle, it is possible to write down an expression for the strain energy per structural unit in the following manner:

(7) $$U=\frac{1}{V}W$$

where $V=L_{x}L_{y}L_{z}$ is the volume of the unit-cell. This energy expression can be further used to determine the Young’s moduli along the main axial directions. More specifically, similarly to the approaches presented in other studies [66,67], it can be calculated as:

(8) $$E_{i}=\frac{2U}{\varepsilon_{i}^{2}}=\frac{12L_{i}^{2}}{L_{x}L_{y}L_{z}}K_{h}{\left(\frac{dL_{i}}{d\theta }\right)}^{-2}$$

where *i* = *x*, *y* or *z*. Thus, the respective Young’s moduli can be written down as follows:

(9) $$E_{x}=E_{y}=\frac{3K_{h}{\left(l_{a}^{2}+l_{b}^{2}-2l_{a}l_{b}\cos\left(\theta \right)\right)}^{3.5}}{l_{a}^{2}l_{b}^{2}{\left(l_{a}l_{b}\sqrt{l_{a}^{2}+l_{b}^{2}-2l_{a}l_{b}\cos\left(\theta \right)}\sin^{2}\left(\theta \right)-{\left(l_{a}^{2}+l_{b}^{2}-2l_{a}l_{b}\cos\left(\theta \right)\right)}^{1.5}\cos\left(\theta \right)\right)}^{2}}$$

(10) $$E_{z}=\frac{12K_{h}{\left(l_{a}^{2}+l_{b}^{2}-2l_{a}l_{b}\cos\left(\theta \right)\right)}^{2.5}}{l_{a}^{2}l_{b}^{2}{\left(2l_{a}l_{b}\sin\left(\theta \right)+l_{w}\sqrt{l_{a}^{2}+l_{b}^{2}-2l_{a}l_{b}\cos\left(\theta \right)}\right)}^{2}\sin^{2}\left(\theta \right)}\;.$$

Once the Poisson’s ratios and Young’s moduli in the axial directions are known, it is possible to construct the compliance matrix for the considered system which can later be utilised in order to determine expressions defining its linear compressibilities. However, it is important to first emphasise the fact that in the assumed limit of $l_{w}\to 0$, this system cannot shear in axial directions. In addition to this, due to its symmetry, it can be classified as being transversely isotropic, which leads to further simplifications; e.g., $E_{x}=E_{y}$. Because of this, the compliance matrix **S**, which in general has 6×6 elements $s_{ij}$ (i,j=1,2,…,6), will have only nine non-zero elements, where i,j=1,2,3. More specifically, it can be defined as follows:

(11) $$S=\left[\begin{array}{cccccc} \frac{1}{E_{x}} & -\frac{\nu_{xy}}{E_{x}} & -\frac{\nu_{zx}}{E_{z}} & 0 & 0 & 0\\ -\frac{\nu_{xy}}{E_{x}} & \frac{1}{E_{x}} & -\frac{\nu_{zx}}{E_{z}} & 0 & 0 & 0\\ -\frac{\nu_{xz}}{E_{x}} & -\frac{\nu_{xz}}{E_{x}} & \frac{1}{E_{z}} & 0 & 0 & 0\\ 0 & 0 & 0 & 0 & 0 & 0\\ 0 & 0 & 0 & 0 & 0 & 0\\ 0 & 0 & 0 & 0 & 0 & 0 \end{array}\right].$$

In view of the above, linear compressibility along axial directions can be calculated using the following expression:

(12) $$\beta_{i}=s_{i1}+s_{i2}+s_{i3}$$

where *i* = 1, 2 or 3. After substituting elements from the compliance matrix into the above formula, it is possible to write down the following equations:

(13) $$\beta_{x}=\frac{1}{E_{x}}-\frac{\nu_{xy}}{E_{x}}-\frac{\nu_{zx}}{E_{z}}\;\;\;\beta_{y}=\frac{1}{E_{x}}-\frac{\nu_{xy}}{E_{x}}-\frac{\nu_{zx}}{E_{z}}\;\;\;\beta_{z}=\frac{1}{E_{z}}-2\frac{\nu_{xz}}{E_{x}}$$

Finally, the Poisson’s ratio and linear compressibilities of the considered system measured for an arbitrary direction in xy, xz and yz planes can be found by means of standard transformations described by Nye [65] and expressions defined above.

### 2.4. Prototype

In addition to theoretical results, in this work, we want to also verify the potential of the considered system to exhibit auxetic behaviour by means of the experiment. In order to do this, we are going to use the prototype presented in Figure 1b where its linear dimensions are described in the Parameters section. It is important to note that all of its structural elements, similarly to the assumptions made in the case of the theoretical model, are rigid, and their shapes remain the same during the deformation process. This stems from the fact that all components constituting the experimental prototype were prepared by means of the FDM 3D printer utilising the PLA material. Furthermore, it is important to note that the only mechanism responsible for the deformation of the considered prototype corresponds to the hinging of respective ligaments connected to each other in a pin-jointed manner by means of bolts and screws.

### 2.5. Parameters

To generate the results presented in this work we considered systems with $l_{a}=7$ cm, $l_{b}=3$ cm and $l_{w}=0$ cm. In addition to that, in instances where we are referring to the variation in the $l_{a}/l_{b}$ ratio, $l_{a}$ was kept constant. It is also important to remember that the Poisson’s ratio, which is the main mechanical property investigated in this study, does not depend on the absolute dimensions of the system but only on the ratio of different parameters used to describe the unit-cell. Additionally, in order to determine the linear compressibility of the system, the stiffness constant, $K_{h}$, was set arbitrarily to one, as the magnitude of this factor was not relevant when determining whether it assumes positive or negative values for specific loading directions.

In the case of the experimental prototype, the parameters used in order to design it were set to be the following: $l_{a}=7$ cm, $l_{b}=3$ cm and $l_{w}=3$ cm.

## 3. Results and Discussion

In order to asses the potential of the considered system to exhibit unusual mechanical properties, we are going to consider configurations corresponding to the two characteristic phases of the system where $\theta <\theta_{0}$ and $\theta >\theta_{0}$. In order to do this, two arbitrary values of the θ angle that satisfy this condition, i.e., $\theta =30^{\circ }$ and $\theta =140^{\circ }$, were selected.

**Figure 4:**
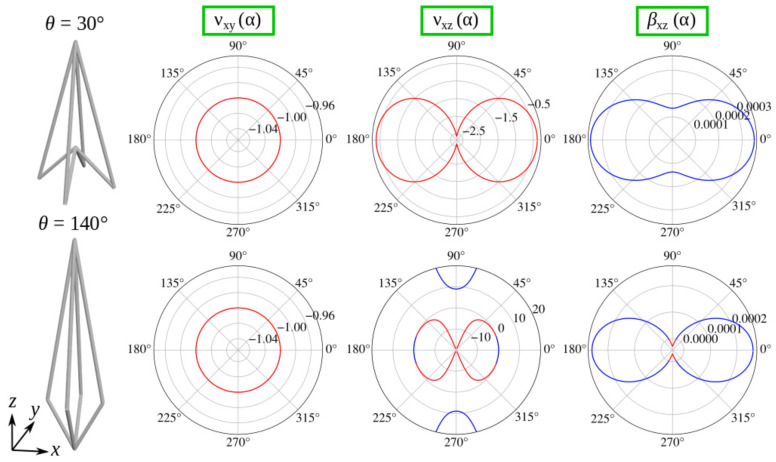
Poisson’s ratio and linear compressibility of two specific configurations of the considered system, where $\theta =30^{\circ }$ ($\theta <\theta_{0}$) and $\theta =140^{\circ }$ ($\theta >\theta_{0}$). Blue and red lines indicate positive and negative values of a given mechanical property respectively. For all of the results, $l_{a}/l_{b}=7/2$.

As shown in Figure 4, loading in an arbitrary direction in the xy plane results in a negative Poisson’s ratio equal to −1. This in-plane isotropic auxetic behaviour can be observed in both situations where $\theta <\theta_{0}$ and $\theta >\theta_{0}$, which is indicative of the fact that such a characteristic is not expected to change as the system deforms. It is also important to emphasise that the Poisson’s ratio equal to −1 is the lower limit of the permissible range of values that an isotropic system can assume [68].

The xz plane was also found to have interesting properties. As shown in Figure 4, the Poisson’s ratio of the system with $\theta <\theta_{0}$ has negative values irrespective of the loading direction. This is a very promising result as it indicates that potential future applications utilising the considered mechanical metamaterial in some configurations would be able to exhibit highly desired auxetic behaviour in this particular plane for any direction in which the mechanical deformation would be applied. On the other hand, this is clearly not the case for configurations where $\theta >\theta_{0}$. For a broad range of possible loading directions in the xz plane, the considered system, similarly to the former case, exhibits auxetic behaviour. However, there are also directions in which the Poisson’s ratio assumes positive values. In fact, there are even loading directions where the Poisson’s ratio has very large positive values; such behaviour, as discussed in other studies [32], can be indicative of other unusual mechanical properties. It is also important to emphasise the fact that due to the symmetry of the system, properties in the yz plane must be identical to those in the xz plane. Furthermore, one can note that results corresponding to the Poisson’s ratios $\nu_{zy}\left(\alpha \right)$ and $\nu_{xz}\left(\alpha \right)$ are almost identical to $\nu_{yz}\left(\alpha \right)$ and $\nu_{zx}\left(\alpha \right)$ respectively, with the only difference being the fact that they would be rotated by $90^{\circ }$ in terms of the values of α.

As shown in Figure 4, the linear compressibility of a system with $\theta <\theta_{0}$, measured for an arbitrary direction in the xz or yz plane, assumes positive values which should be expected considering the negative Poisson’s ratio properties of the system in these planes. However, the linear compressibility of a system where $\theta >\theta_{0}$ manifests a more complex behaviour in the same planes. Specifically, the linear compressibility assumes positive values for a broad range of angles α. However, there are some directions associated with a very high positive Poisson’s ratios where NLC can be observed.

**Figure 5:**
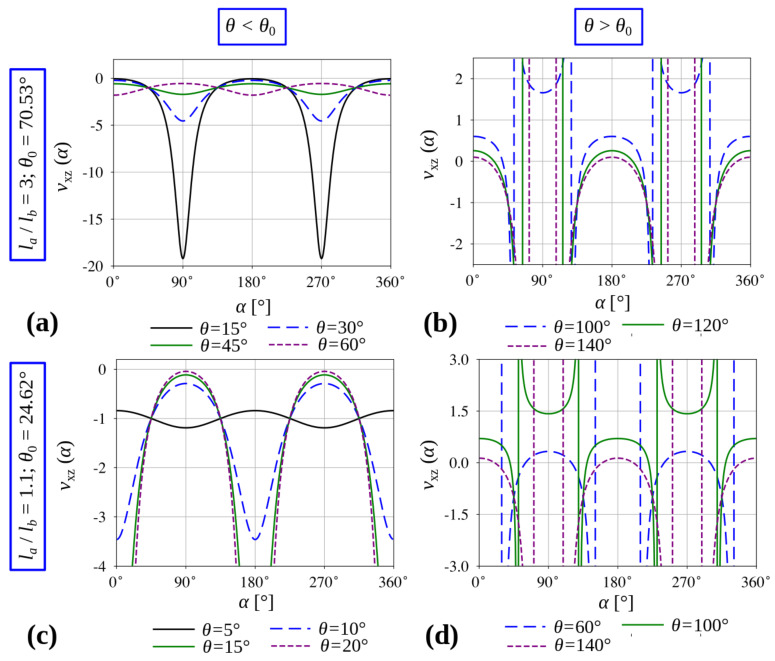
Poisson’s ratio for loading in an arbitrary direction in the xz plane (in the yz plane results are identical) for the system having different ratios of linear dimensions constituting the system $l_{a}/l_{b}$.

It is important to remember that the results portrayed in Figure 4 correspond to a specific system where $l_{a}/l_{b}=7/2$. In view of this, it would be interesting to check how the properties of the considered mechanical metamaterial would change should it be constructed from ligaments having a different ratio of linear dimensions. In addition to this, it would also be interesting to determine how these properties change with the value of θ. Such analysis is provided in Figure 5.

As shown in Figure 5a,c, configurations where $\theta <\theta_{0}$ always exhibit auxetic behaviour for loading in any arbitrary direction in the xz and yz planes irrespective of the $l_{a}/l_{b}$ ratio and the value of θ, given that it lies within the aforementioned interval. Furthermore, upon closer analysis of the results, one can note that the system associated with a relatively large $l_{a}/l_{b}$ ratio where θ is very small (see Figure 5a), has characteristic peaks of a very negative Poisson’s ratio for two specific and mutually opposite loading directions. However, the same system shows a Poisson’s ratio close to zero for other loading directions. In fact, this behaviour changes gradually upon increasing the value of θ; i.e., the aforementioned peaks start diminishing, while at the same time in other loading directions, the system assumes more negative values of the Poisson’s ratio. It is also worth mentioning that, as shown in Figure 5c, the system having ligaments $l_{a}$ and $l_{b}$ of a comparable length (e.g., $l_{a}/l_{b}=1.1$) exhibits a similar, but to a certain extent opposite behaviour to trends portrayed in Figure 5a. More specifically, the peaks of a low Poisson’s ratio occur for values of θ close to $\theta_{0}$, instead of θ→0, as was formerly the case.

**Figure 6:**
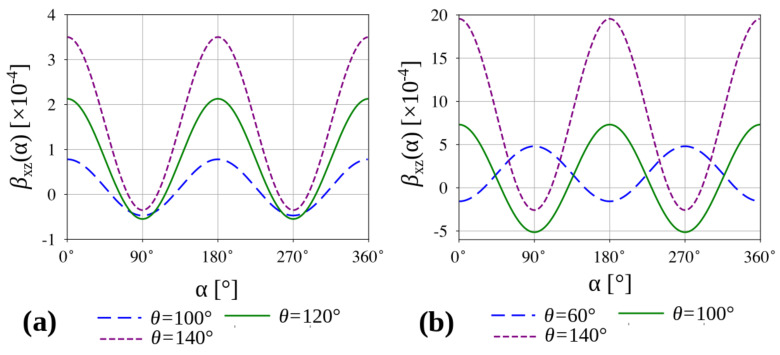
Linear compressibility measured in the xz plane (the same results would be obtained in the yz plane). Panels show (**a**) linear compressibility of the system where $l_{a}/l_{b}=3$ and (**b**) linear compressibility of the system correspond to the $l_{a}/l_{b}=1.1$ ratio.

According to Figure 5b,d and Figure 6, configurations where $\theta >\theta_{0}$ are capable of exhibiting auxetic behaviour or NLC in the xz and yz planes depend on the specific loading direction. Nevertheless, irrespective of the value of the $l_{a}/l_{b}$ ratio, both of these unusual mechanical properties occur only for some loading directions and it is never the case that the system exhibits solely auxetic behaviour or conversely NLC.

**Figure 7:**
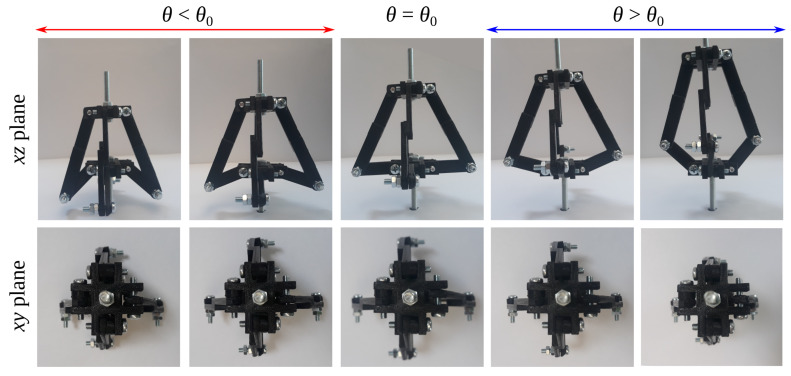
Deformation of the prototype corresponding to $\theta_{0}=64.62^{\circ }$ presented from the perspective of the xz and xy planes.

In addition to theoretical results, which are the main focus of this work, one can also validate the potential of the considered system to exhibit auxetic behaviour through experimental means. In order to do this, it is possible to utilise a prototype like the one presented in Figure 1b. According to Figure 7, it is clear to see that in the xz plane, the considered system is capable of exhibiting auxetic behaviour for loading in the *z* direction as long as $\theta <\theta_{0}$. On the other hand, for configurations associated with $\theta >\theta_{0}$, its lateral dimension decreases as it is being loaded uniaxially along the *z* axis, which is indicative of a positive Poisson’s ratio. It is also important to emphasise the fact that the considered prototype exhibits auxetic behaviour for a broad range of loading directions other than the axial associated with the *z* axis. To better visualise it, one can consider the use of the theoretical model proposed in this work with geometric parameters matching the experimental prototypes. In fact, as shown in Appendix B, where such additional analysis is provided, the model with parameters identical to the experimental prototype exhibits the same trends as is the case for the formerly discussed results. However, it is important to remember that in this particular situation, $l_{w}>0$, which therefore, as discussed in the Methods section, does not satisfy the assumption made by the model, so one expects that the mechanical properties manifested by structures composed of multiple structural units in real life will not exactly match those predicted by the theoretical model. Furthermore, as shown in Figure 7, the cross-section of the experimental prototype in the xy plane resembles a square irrespective of the stage of the mechanical deformation. This in turn, similarly to the former analytical predictions, suggests that the system can exhibit in-plane isotropic auxetic behaviour corresponding to a Poisson’s ratio equal to −1 for all loading directions in this particular plane. This result can be also observed from the theoretical model with dimensions corresponding to those used in the experimental prototype, as shown in Appendix B.

This work is of great importance, as it has shown that the considered mechanical metamaterial is capable of exhibiting a very versatile mechanical behaviour depending on its geometric parameters and assumed configuration. More specifically, as discussed for specific values of θ ($\theta <\theta_{0}$) the system always exhibits auxetic behaviour for an arbitrary loading direction in the xz and yz planes. It is also capable of manifesting an in-plane isotropic auxetic behaviour for loading in the xy plane irrespective of other parameters considered in this work. In addition to that, there are also configurations associated with $\theta >\theta_{0}$ where the considered mechanical metamaterial can exhibit either auxetic behaviour or NLC. All of this suggests that the analysed system has a great potential to be implemented in industry in materials which exhibit counter-intuitive mechanical properties upon being subjected to external stimuli from very different directions. Such behaviour is essential in many applications where it would be difficult to adjust the spatial orientation of the system to a given type of stimulus. In view of this, it is hoped that the results discussed in this work will lead to further studies related to the potential of the system described in this work to be implemented in efficient protective devices where it is very difficult to predict the direction of the external cause of the mechanical deformation. This stems from the fact that protective devices require an enhanced impact resistance which is often attributed to auxetic mechanical metamaterials [8]. However, the majority of such materials are either two-dimensional, which normally does not allow them to exhibit negative Poisson’s ratio in directions which do not correspond to the in-plane deformation, or are capable of exhibiting auxetic behaviour only for very specific loading directions, which significantly reduces their applicability. Thus, three-dimensional mechanical metamaterials capable of exhibiting auxetic behaviour for a very broad range of loading directions, as is the case in this work, are expected to act as very efficient protective devices that do not have to be reoriented in space in order to maintain their protective properties. Furthermore, as discussed in [28], the unusual mechanical properties of this system can also be used in order to design smart mechanical metamaterials utilising mutually interacting magnetic inclusions, which, depending on the scale of the structure, could potentially lead to the design of magneto-mechanical refrigerators [69] or efficient damping devices [9].

## 4. Conclusions

In this work, it was shown that a particular 3D mechanical metamaterial composed of arrowhead-like structural units can exhibit very versatile counter-intuitive mechanical behaviour for a myriad of loading directions. More specifically, it was shown that it is capable of exibiting isotropic auxetic behaviour for an arbitrary loading direction in the xy plane. In addition to this, it was found to always manifest a negative Poisson’s ratio for loading in any direction in the xz or yz plane when $\theta <\theta_{0}$. In fact, it can also exhibit a very interesting mechanical behaviour when $\theta >\theta_{0}$; i.e., it can exhibit either auxetic behaviour or NLC in the xz and yz planes depending on which deformation direction is selected. All of these properties make it capable of exhibiting a desired mechanical response upon being subjected to an external stimulus applied from different spatial directions. In view of this, it is hoped that the considered mechanical metamaterial can be applied in applications such as protective devices, where properties inherent to auxetic materials are needed and it is very difficult to predict the direction in which the system is going to be deformed.

## Figures and Tables

**Figure A2:**
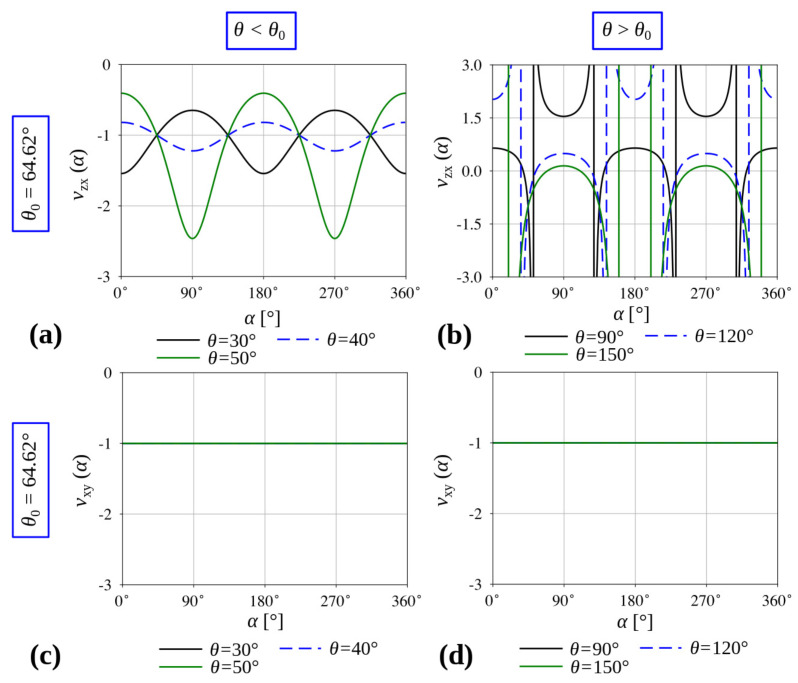
Panels show analytical results corresponding to a system with parameters matching those used in the experiment for loading in (**a**,**b**) xz plane and (**c**,**d**) xy plane.

## Abbreviations

The following abbreviations are used in this manuscript:

NLC	Negative Linear Compressibility

## Appendix A. Design of The Prototype

In order to better explain how the considered model could be turned into the experimental prototype composed of numerous structural units, we show details of how structural units like the one shown in the main text could be connected to each other. Each of the structural units shown in Figure A1 is, in terms of its design, identical to the 3D-printed unit-cell presented in the main text. It is also worth noting that cylindrical apertures present in the provided diagram indicate places where bolts acting as axes of rotation for ligaments should be inserted upon assembling the system.

## Appendix B. Theoretical Results Corresponding to Parameters Used in the Experiment

In this appendix, we provide analytical results describing Poisson’s ratio for loading in the arbitrary direction in the xz and xy planes for a system with geometric parameters matching those used in the case of the design of the experimental prototype shown in Figure 1b.
